# Reply: Beyond Symmetry and Expansion: Coaxiality and Its Impact on Long-Term TAVR Outcomes

**DOI:** 10.1016/j.jacadv.2025.102181

**Published:** 2025-09-18

**Authors:** Domenico Angellotti, Annette Maznyczka, Jonas Lanz, Fabien Praz, Thomas Pilgrim

**Affiliations:** a Department of Cardiology, Bern University Hospital, Bern, Switzerland; b Department of Advanced Biomedical Science, University of Naples Federico II, Naples, Italy; c Department of Cardiology, Leeds Teaching Hospitals NHS Trust, Leeds, United Kingdom

**Figure 1:**
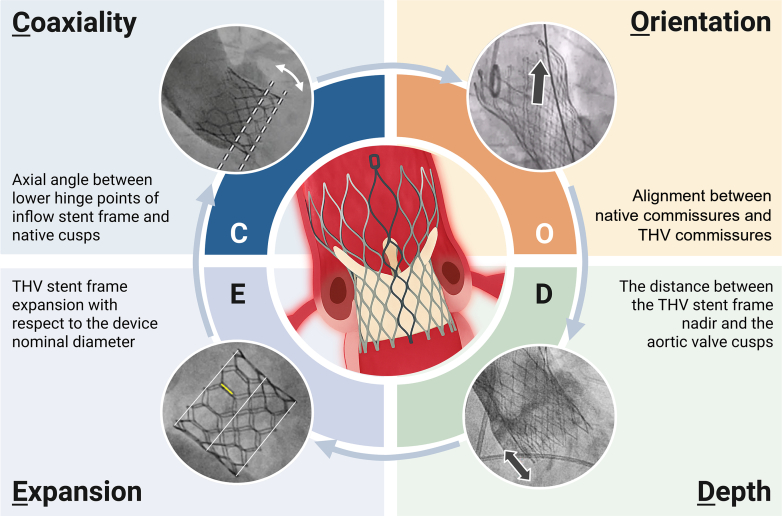
**The CODE Framework for Transcatheter Aortic Valve Optimization** TAVR = transcatheter aortic valve replacement; THV = transcatheter heart valve.

We thank Dr Skalidis and colleagues for their interest in our analysis on transcatheter aortic valve replacement (TAVR) coaxiality, which underscores the association between noncoaxial transcatheter heart valve (THV) deployment and suboptimal valve performance.^1^ We concur that modifiable procedural factors influencing valve performance merit close attention. Notably, intraprocedural fluoroscopic assessment serves as a valuable tool for identifying suboptimal THV deployment. The CODE framework (Coaxiality, Orientation, Depth of implantation, and Expansion of THV) integrates key parameters for optimizing TAVR under fluoroscopy guidance and complements thorough preprocedural assessment of anatomic suitability as well as comprehensive postprocedural management (Figure 1). Specifically, it allows for evaluation of the following key elements, which are critical for optimizing TAVR outcomes.

Noncoaxial THV implantation, defined as an axial angle ≥4.8° between the native annulus and the prosthesis frame, may predict impaired early device safety and an increased risk of reintervention at 1-year follow-up (HR: 3.47; 95% CI: 1.26-9.54).^1^

Commissural alignment is determined by the angular relationship between native and THV commissures. Obtaining proper commissural alignment during TAVR facilitates future coronary access, improves hemodynamics, and preserves the option to perform leaflet laceration in case of redo-TAVR.^2^

A deeper THV implant and closer proximity of the stent frame to the membranous septum predict an increased risk of permanent pacemaker implantation.^3^ Conversely, a higher THV implantation affects the height of the neo-skirt in future TAV-in-TAV implantations.

Underexpansion of the THV stent frame has been associated with paravalvular leak, hypoattenuated leaflet thickening, and more recently even with an increased risk of a composite of death, stroke, or rehospitalization.^4^

Moving forward, it is essential to navigate the CODE pitfalls more effectively. For example, wire stiffness and position, pre-dilatation, and steerable catheters may facilitate coaxiality; orientation markers or locators can assist in achieving commissural alignment; accurate imaging and stable THV release mechanisms improve THV positioning; and adequate post-dilation effectively mitigates underexpansion. Moreover, we need to develop strategies to prevent known nonmodifiable factors of suboptimal THV performance, such as stent frame asymmetry and THV eccentricity, in order to continuously improve TAVR outcomes.^5^
